# Rapid CSF Molecular Diagnosis of *Listeria monocytogenes* Meningoencephalitis in an Elderly Immunocompetent Patient: A Case Report

**DOI:** 10.3390/diagnostics16193162

**Published:** 2026-09-29

**Authors:** Ákos Vince Andrejkovits, Róbert Ladó, Adrian Vlad Pop, Răzvan Lucian Coșeriu, Camelia Vintilă, Adrian Man, Anca Meda Văsieșiu, Elena Cristina Gîrbovan

**Affiliations:** 1 Department of Infectious Diseases, George Emil Palade University of Medicine, Pharmacy, Science and Technology of Târgu Mureș, 540142 Târgu Mureș, Romania; akos.andrejkovits@umfst.ro (Á.V.A.); pop.adrian-vlad.25@stud.umfst.ro (A.V.P.); anca-meda.vasiesiu@umfst.ro (A.M.V.); 2 1st Infectious Disease Clinic, Mureș County Clinical Hospital, 540233 Târgu Mureș, Romania; lado.robert27@gmail.com; 3 Department of Microbiology, George Emil Palade University of Medicine, Pharmacy, Science and Technology of Târgu Mureș, 540142 Târgu Mureș, Romania; lucian-razvan.coseriu@umfst.ro (R.L.C.); camelia.vintila@umfst.ro (C.V.); adrian.man@umfst.ro (A.M.)

**Keywords:** *L. monocytogenes*, neurolisteriosis, meningoencephalitis, cerebrospinal fluid, molecular diagnosis, FilmArray®

## Abstract

**Background/Objectives: ***Listeria monocytogenes* is an uncommon but potentially life-threatening cause of central nervous system infection, predominantly affecting older adults and immunocompromised patients. Neurolisteriosis may present with nonspecific neurological manifestations, and the absence of classical meningeal signs may delay diagnosis. We report a case of *L. monocytogenes* meningoencephalitis in an elderly patient without known immunosuppression, with rapid cerebrospinal fluid (CSF) molecular testing facilitating early diagnosis and appropriate antimicrobial treatment. **Case presentation:** A 76-year-old man with significant cardiovascular comorbidities was admitted with a 3-day history of fever, headache, nausea, vomiting, fatigue, and gait instability. He was alert on admission, with only mild meningeal signs, and initial cranial computed tomography showed no acute intracranial abnormalities. CSF analysis revealed inflammatory changes. Empirical ceftriaxone therapy was supplemented with vancomycin, followed by the initiation of intravenous ampicillin and gentamicin after molecular identification of *L. monocytogenes*. The diagnosis was subsequently confirmed by CSF culture, while blood cultures remained negative. Clinical and laboratory parameters improved progressively, accompanied by marked improvement in CSF findings on follow-up examination. The patient was discharged in stable condition after 18 days of hospitalization. **Conclusions:** Neurolisteriosis should be considered in elderly patients presenting with fever and neurological symptoms, even in the absence of overt immunosuppression or prominent meningeal signs. This case highlights the diagnostic value of rapid CSF molecular testing, particularly when blood cultures are negative and the initial clinical presentation is nonspecific. Early recognition of *L. monocytogenes* infection and timely initiation of targeted antimicrobial therapy are essential to improving outcomes.

## 1. Introduction

*L. monocytogenes* is a Gram-positive, facultative intracellular rod that is widely distributed in the environment, including soil, surface water, and the gastrointestinal tracts of animals and humans. Its ability to tolerate adverse environmental conditions, including low temperatures, acidic environments, and high salt concentrations, contributes to its persistence in food production and storage environments. Human infections are primarily associated with the consumption of contaminated food. The clinical spectrum ranges from mild, febrile gastroenteritis to invasive and potentially life-threatening infections, including bacteremia, meningitis, and meningoencephalitis [1].

Risk factors predisposing to invasive listeriosis include hematological and other malignancies, solid-organ transplantation, immunosuppressive therapy, Human immunodeficiency virus (HIV) infection, diabetes mellitus, chronic liver disease, and alcohol use disorder, as well as other conditions associated with impaired cell-mediated immunity. The risk of infection increases substantially with advancing age [2,3].

Although listeriosis is an uncommon foodborne infection, invasive disease is associated with substantial morbidity and mortality, particularly among older adults and individuals with underlying risk factors. According to the European Centre for Disease Prevention and Control (ECDC), 2993 confirmed cases of listeriosis were reported across 30 European Union/European Economic Area (EU/EEA) countries in 2023, representing the highest annual number recorded since the beginning of EU/EEA-wide surveillance. Among cases for which hospitalization status was available, 96.6% required hospital admission, highlighting the predominantly severe nature of clinically apparent listeriosis. Furthermore, among patients with a known outcome, the case-fatality rate was approximately 19% [4]. These data highlight the continuing public health importance of invasive listeriosis in Europe.

Central nervous system (CNS) infection, or neurolisteriosis, represents one of the most severe manifestations of invasive listeriosis. The clinical spectrum includes meningitis, meningoencephalitis, rhombencephalitis, and, less frequently, brain abscesses [5]. The mechanisms by which *L. monocytogenes* reaches the CNS are not fully understood, although hematogenous dissemination and retrograde neural spread have been proposed [6].

The diagnosis of neurolisteriosis can be challenging because of its heterogeneous clinical presentation and the frequent absence of classical meningeal signs. In addition to fever, headache, neck stiffness, and altered mental status, patients may present with seizures, focal neurological deficits, cranial nerve involvement, or other nonspecific neurological manifestations. CSF findings are not pathognomonic, and microbiological confirmation may be challenging when culture sensitivity is limited, particularly after antimicrobial exposure. Blood cultures may also be negative, while neuroimaging can be normal or demonstrate nonspecific abnormalities [5]. Consequently, the diagnosis may be delayed, potentially postponing the initiation of appropriate antimicrobial therapy.

Rapid molecular diagnostic methods have emerged as valuable adjuncts in the evaluation of suspected CNS infections. Multiplex nucleic acid amplification panels can simultaneously detect multiple bacterial, viral, and fungal pathogens directly from CSF, potentially reducing the time to etiological diagnosis compared with conventional culture-based methods. In the context of suspected neurolisteriosis, rapid identification of *L. monocytogenes* is particularly relevant because the organism has intrinsic resistance to third-generation cephalosporins, including ceftriaxone and cefotaxime. Current meningitis treatment guidelines recommend the inclusion of intravenous ampicillin or amoxicillin in empirical therapy when *L. monocytogenes* is suspected [7]. Following microbiological confirmation, ampicillin, amoxicillin, and penicillin G are recommended therapeutic options, with the addition of an aminoglycoside considered in selected patients [5].

In this context, rapid and accurate microbiological diagnosis may have important implications for antimicrobial selection and clinical management. We present a case of *L. monocytogenes* meningoencephalitis in an elderly patient with significant cardiovascular comorbidities but no recognized immunosuppressive condition. The case highlights the diagnostic challenges associated with an atypical clinical presentation and negative blood cultures, and emphasizes the potential role of rapid CSF molecular testing in enabling early identification of *L. monocytogenes* and timely initiation of targeted antimicrobial therapy.

## 2. Case Presentation

A 76-year-old man was admitted to the Infectious Diseases Department with a 3-day history of fever, chills, headache, nausea, recurrent vomiting, marked fatigue, and gait instability. His medical history was notable for Braunwald class IIIB unstable angina, ischemic heart disease, New York Heart Association (NYHA) class III heart failure with reduced ejection fraction, moderate mitral and tricuspid regurgitation, permanent atrial fibrillation with a low ventricular response, grade 2 hypertension, previous percutaneous coronary intervention with stent placement, and permanent pacemaker implantation in 2026. Additional comorbidities included hypercholesterolemia, hemorrhoidal disease, and colonic diverticulosis. He had no known drug allergies and reported no smoking or alcohol consumption. He reported occasional coffee consumption. No specific epidemiological exposure was identified from the available history; in particular, there was no documented history of relevant infectious contacts or high-risk food exposure.

The patient initially presented to the Emergency Department. Neurological examination showed mild limitation of cervical spine mobility, which the patient reported as pre-existing. Kernig and Brudzinski signs were negative. No cranial nerve, motor, sensory, or coordination abnormalities were found, and osteotendinous reflexes were preserved. He was conscious and fully oriented, with mild photophobia. Because prominent meningeal signs were absent and the patient had taken oral anticoagulant therapy on the day of evaluation, lumbar puncture was initially deferred.

Cranial computed tomography (CT) showed no acute intracranial abnormalities. Chest radiography revealed no focal pulmonary consolidation or pleural effusion. Initial laboratory investigations demonstrated mild leukocytosis (10.76 × 10^3^/µL) with marked neutrophilia (93%), mild hyponatremia (132 mmol/L), and a pronounced systemic inflammatory response. The procalcitonin concentration was 0.16 ng/mL, and renal function was preserved. The evolution of systemic inflammatory and laboratory parameters during hospitalization is presented in Table 1.

Following transfer to the Infectious Diseases Department, the patient remained in poor general condition but was conscious and fully oriented. His temperature was 38.7 °C, blood pressure was 144/98 mmHg, heart rate was 89 beats/min, and oxygen saturation was 96% on room air. Neurological examination at that time demonstrated bradylalia, neck stiffness, mildly positive Kernig I and II signs, and a positive Romberg test. No sensory deficits, coordination abnormalities, or cranial nerve involvement were identified. Based on the evolving neurological findings, persistent fever, and marked inflammatory response, meningoencephalitis was suspected, and empirical intravenous ceftriaxone was initiated. Dexamethasone, mannitol, symptomatic therapy, and intravenous fluids were administered concomitantly.

On the second hospital day, lumbar puncture was performed. The CSF was clear and demonstrated pleocytosis with a total nucleated cell count of 320 cells/µL, comprising 55% neutrophils and 45% lymphocytes. The CSF protein concentration was elevated at 158.6 mg/dL, while the glucose concentration was decreased at 28 mg/dL, compared with a concomitant serum glucose concentration of 105 mg/dL. The CSF chloride concentration was 113 mmol/L.

Given the inflammatory CSF findings, intravenous vancomycin was added to the empirical ceftriaxone treatment while awaiting microbiological results.

The CSF sample was analyzed using the BioFire® FilmArray® Meningitis/Encephalitis (ME) Panel v1.4 (BioFire Diagnostics, LLC, Salt Lake City, UT, USA), which detected *L. monocytogenes* (Figure 1). Intravenous ampicillin and gentamicin were subsequently initiated.

The diagnosis was subsequently confirmed by CSF culture. After 18 h of incubation, small, smooth, round, translucent colonies measuring approximately 1–2 mm in diameter and surrounded by a narrow zone of β-haemolysis were observed on Columbia agar (Figure 2). No relevant growth was observed on the other culture media used. The isolate was identified as *L. monocytogenes* by matrix-assisted laser desorption/ionization time-of-flight mass spectrometry (MALDI-TOF MS) using the MALDI Biotyper Sirius system (Bruker, Billerica, MA, USA), with an identification score of 2.34 (Figure 3).

Antimicrobial susceptibility testing was performed using the Kirby–Bauer disk diffusion method. The isolate was susceptible to ampicillin, meropenem, and trimethoprim-sulfamethoxazole according to the applicable European Committee on Antimicrobial Susceptibility Testing (EUCAST) interpretive criteria. The isolate was susceptible to ampicillin, meropenem, and trimethoprim–sulfamethoxazole. MIC testing was not performed. Aerobic and anaerobic blood cultures obtained during the diagnostic evaluation remained negative. Urinary antigen tests for *Streptococcus pneumoniae* and *Legionella pneumophila* were also negative.

Following identification of *L. monocytogenes* and confirmation of ampicillin susceptibility, targeted intravenous ampicillin therapy was initiated in combination with gentamicin. Ampicillin was administered from hospital day 3 until discharge, while gentamicin was administered for 6 days. Adjunctive dexamethasone and mannitol were continued initially, with dexamethasone subsequently tapered during hospitalization. Supportive treatment included intravenous fluid therapy and symptomatic management, including baclofen for persistent hiccups.

Because of the reduced CSF chloride concentration, tuberculous meningitis was also considered in the differential diagnosis. CSF testing using the GeneXpert (Cepheid, Sunnyvale, CA, USA) assay did not detect *Mycobacterium tuberculosis*, and microscopic examination for acid-fast bacilli was negative.

During hospitalization, the patient became afebrile, and systemic inflammatory parameters progressively decreased. Nevertheless, neurological abnormalities persisted beyond the resolution of fever and systemic inflammation. The patient remained brady psychic and developed persistent hiccups and increasing difficulty walking. Neurological reassessment demonstrated a persistently positive Romberg test and reduced lower-limb osteotendinous reflexes, which were difficult to elicit.

Because of the persistence and evolution of the neurological manifestations, further neurological evaluation was undertaken. Contrast-enhanced brain magnetic resonance imaging (MRI) performed on hospital day 11 demonstrated several small periventricular microvascular white-matter lesions and periventricular leukoaraiosis. No pathological intraparenchymal or leptomeningeal contrast enhancement, diffusion restriction, brainstem or cerebellar abnormalities, or other acute intracranial pathology was identified (Figure 4).

The patient subsequently demonstrated progressive clinical improvement with continued targeted antimicrobial and supportive therapy. Neurological manifestations gradually resolved, and fever remained absent. Given the persistence of neurological symptoms during the course of treatment, a follow-up lumbar puncture was performed on hospital day 16 to assess the evolution of the CSF abnormalities. The CSF remained clear and showed marked improvement in inflammatory parameters, with a total cell count of 68 cells/µL, protein concentration of 53.5 mg/dL, glucose concentration of 63 mg/dL, and chloride concentration of 117 mmol/L. Repeat microbiological examination showed no microbial growth. The CSF findings at diagnosis and follow-up are summarized in Table 2.

Overall, the clinical presentation, inflammatory CSF profile, positive CSF molecular assay, and subsequent culture confirmation established the diagnosis of *L. monocytogenes* meningoencephalitis. A timeline of the patient’s clinical course, diagnostic investigations, microbiological results, and antimicrobial treatment is presented in Table 3. The patient was discharged on hospital day 18 with clinical improvement, no fever, and stable hemodynamic and respiratory status. Intravenous antimicrobial therapy was completed during hospitalization, and oral amoxicillin 1 g three times daily was prescribed for an additional 6 days after discharge.

## 3. Discussion

This case illustrates several diagnostic and therapeutic challenges associated with *L. monocytogenes* CNS infection. Neurolisteriosis predominantly affects older adults and individuals with impaired cell-mediated immunity, but clinical manifestations are heterogeneous and may be nonspecific, potentially delaying etiological diagnosis and appropriate antimicrobial therapy [8,9,10]. In the present case, the initial absence of prominent meningeal signs, the lack of acute abnormalities on cranial CT, and the initial deferral of lumbar puncture because of recent anticoagulant therapy contributed to diagnostic uncertainty.

At 76 years of age, our patient belonged to a recognized high-risk group for invasive listeriosis. Amaya-Villar et al. reported a median age of 69 years among adults with neurolisteriosis [11]. Notably, our patient had no known immunosuppressive condition and was not receiving immunosuppressive or corticosteroid therapy. Fasting blood glucose levels were normal, although HbA1c was not assessed, and no HIV testing was performed during hospitalization. The patient reported no alcohol consumption, and his chronic medications did not include immunosuppressive agents.

Similar observations have been reported by Tago et al., who described neurolisteriosis in an elderly patient without classical immunosuppressive conditions [12].

In the absence of documented immunosuppression or other major predisposing conditions, advanced age may have represented an important risk factor for *L. monocytogenes* infection in our patient. These observations highlight that the absence of a known conventional immunosuppressive condition should not substantially lower clinical suspicion for neurolisteriosis in older patients presenting with compatible neurological symptoms.

The clinical presentation of neurolisteriosis can differ from that of more typical bacterial meningitis. In a study of 30 adults with *L. monocytogenes* meningitis, Brouwer et al. reported the classical triad of fever, neck stiffness, and altered mental status in only 43% of patients [10]. Amaya-Villar et al. similarly reported that the complete triad was present in approximately half of patients with neurolisteriosis [11]. In our patient, the initial neurological examination was relatively nonspecific: he was conscious and fully oriented, and Kernig and Brudzinski signs were initially negative. Mild photophobia and pre-existing limitation of cervical mobility were the only potentially suggestive findings. More definite neurological abnormalities, including bradylalia, neck stiffness, positive Kernig signs, and a positive Romberg test, became apparent following admission. This evolution illustrates that the neurological examination may change over the early course of neurolisteriosis and that the absence of marked meningeal signs at initial assessment does not exclude CNS infection [8,10,11,12].

Neuroimaging findings in neurolisteriosis are also variable and may be normal or nonspecific. In the present case, the initial cranial CT showed no acute abnormalities. Subsequent contrast-enhanced MRI demonstrated only chronic-appearing microvascular white-matter changes and periventricular leukoaraiosis, without leptomeningeal or intraparenchymal enhancement, diffusion restriction, or brainstem or cerebellar abnormalities. Amaya-Villar et al. reported normal cranial CT findings in 77% of patients with neurolisteriosis [11], whereas Charlier et al. described heterogeneous imaging abnormalities among 71 confirmed cases [13]. The absence of characteristic neuroimaging abnormalities therefore should not be interpreted as evidence against neurolisteriosis when the clinical and CSF findings remain suggestive.

The microbiological findings in our patient emphasize the importance of CSF-based testing in suspected neurolisteriosis. The CSF BioFire® FilmArray® Meningitis/Encephalitis Panel rapidly detected *L. monocytogenes*, and the result was subsequently confirmed by CSF culture and MALDI-TOF mass spectrometry. In contrast, both aerobic and anaerobic blood cultures remained negative. Blood culture positivity in neurolisteriosis is variable, ranging from 63% in the study by Amaya-Villar et al. [11] to 68.5% in the 16-year Hungarian cohort reported by Kiss et al. [14], whereas CSF cultures were positive in 87.3% of cases in the latter study. These findings indicate that negative blood cultures do not exclude *L. monocytogenes* CNS infection and support the importance of direct CSF microbiological investigation in patients with compatible clinical and laboratory findings.

Rapid molecular testing can provide etiological information considerably earlier than conventional culture. The BioFire® FilmArray® Meningitis/Encephalitis Panel was developed as a multiplex molecular assay capable of detecting multiple bacterial, viral, and fungal pathogens directly from CSF [15]. Subsequent systematic reviews and diagnostic accuracy studies have demonstrated high overall specificity and useful clinical performance, although sensitivity varies among individual pathogens and may be suboptimal for some organisms, including *L. monocytogenes* [16,17]. Therefore, a negative molecular result should not necessarily exclude infection when clinical suspicion remains high. In the present case, the positive FilmArray® result was particularly valuable because it provided a rapid etiological diagnosis that was subsequently independently confirmed by CSF culture and MALDI-TOF MS. This combination of rapid molecular detection and conventional microbiological confirmation strengthened diagnostic certainty while allowing earlier modification of empirical antimicrobial therapy.

The therapeutic implications of rapid identification are particularly important because *L. monocytogenes* is intrinsically resistant to third-generation cephalosporins, including ceftriaxone and cefotaxime [5,7]. Our patient initially received empirical ceftriaxone, which was supplemented with vancomycin because of the inflammatory CSF profile. Once *L. monocytogenes* was identified by CSF PCR, ceftriaxone and vancomycin were discontinued, and targeted treatment with intravenous ampicillin was initiated. The CSF isolate was subsequently confirmed by culture and was susceptible to ampicillin. Gentamicin was administered in combination with ampicillin based on the reported synergistic and bactericidal activity of this combination against *L. monocytogenes*, although the clinical benefit of combination therapy over ampicillin alone remains uncertain. Similar diagnostic and therapeutic evolution, from empirical cephalosporin therapy to targeted treatment following microbiological identification, has been described in previous reports [9]. This sequence highlights the potential clinical consequences of delayed recognition of *L. monocytogenes*, as an empirically appropriate regimen for many causes of bacterial meningitis may provide inadequate coverage for this pathogen.

An additional feature of this case was the persistence of neurological manifestations despite progressive resolution of fever and systemic inflammation. The patient developed persistent hiccups, bradypsychia, gait impairment, and reduced lower-limb reflexes during the early treatment period, prompting further neurological assessment and MRI. The absence of acute structural abnormalities on MRI and the subsequent gradual neurological improvement suggested a prolonged clinical recovery rather than a new structural CNS complication. The follow-up lumbar puncture on hospital day 16 demonstrated substantial improvement in CSF parameters, including a reduction in the total nucleated cell count from 320 to 68 cells/µL and in CSF protein from 158.6 to 53.5 mg/dL, together with normalization of CSF glucose. Repeat microbiological examination was negative. The dissociation between improvement in systemic and CSF inflammatory parameters and the slower resolution of neurological manifestations is clinically relevant, as neurological recovery in CNS infection may lag behind microbiological and inflammatory improvement.

The use of corticosteroids in neurolisteriosis remains controversial, and there is no established indication for their routine use. Corticosteroids may be considered in bacterial meningitis when there is significant meningeal inflammation or concern for complications related to cerebral edema or increased intracranial pressure; however, their benefit is best established in selected cases of pneumococcal meningitis. In *L. monocytogenes* meningitis, evidence supporting adjunctive corticosteroid therapy is limited, and concerns have been raised that corticosteroid-induced immunosuppression may impair bacterial clearance. Therefore, their use should be individualized according to the clinical presentation and suspected complications. In our patient, dexamethasone was initially administered as adjunctive therapy for suspected bacterial meningitis. Because neurological symptoms persisted, it was continued due to clinical concern for possible brainstem involvement. Brain MRI subsequently showed no evidence of brainstem involvement or other acute intracranial pathology, and dexamethasone was gradually tapered.

The differential diagnosis in this case also included tuberculous meningitis because of the reduced CSF chloride concentration. However, CSF GeneXpert testing and acid-fast bacilli microscopy were negative. Importantly, reduced CSF chloride is nonspecific and should not be interpreted in isolation as evidence of tuberculous meningitis. In the present case, the positive *L. monocytogenes* molecular assay, subsequent CSF culture confirmation, compatible inflammatory CSF profile, and clinical response to targeted antimicrobial therapy provided a coherent explanation for the patient’s presentation.

Neuroimaging findings in neurolisteriosis can be variable and may include meningeal enhancement, parenchymal lesions, abscesses, brainstem involvement, and, in some cases, hydrocephalus. Hydrocephalus may occur as a complication of *L. monocytogenes* meningitis, reflecting impaired cerebrospinal fluid circulation in the setting of meningeal inflammation. A recent case report described *L. monocytogenes* meningitis complicated by hydrocephalus in a patient with Sjögren’s syndrome [18]. In our patient, brain MRI did not demonstrate hydrocephalus, leptomeningeal or intraparenchymal enhancement, diffusion restriction, or brainstem or cerebellar abnormalities. The absence of these findings illustrates that neurolisteriosis may occur without characteristic acute neuroimaging abnormalities despite persistent neurological manifestations.

The favorable clinical outcome in our patient is notable in view of the substantial mortality associated with invasive listeriosis. Kiss et al. reported a mortality rate of 27% in their 16-year cohort of invasive listeriosis [14]. Although the patient required prolonged hospitalization and experienced persistent neurological manifestations during the early treatment course, he ultimately became afebrile, showed progressive neurological recovery, demonstrated marked improvement in CSF parameters, and was discharged in stable condition after 18 days, with continuation of oral amoxicillin after discharge.

This case has several limitations. First, as a single case report, it cannot establish the diagnostic sensitivity or clinical superiority of multiplex molecular testing over conventional microbiological methods. Second, no specific food exposure or epidemiological source could be identified, and therefore the source of infection remains uncertain. Third, the patient’s multiple cardiovascular comorbidities and advanced age represent potential contributors to his clinical course, making it difficult to attribute all neurological manifestations exclusively to the CNS infection. Nevertheless, the simultaneous positive CSF molecular assay and CSF culture, together with the compatible clinical and laboratory findings and subsequent response to targeted therapy, provide strong evidence supporting the diagnosis.

Overall, this case emphasizes that neurolisteriosis should remain in the differential diagnosis of CNS infection in older adults, even when classical meningeal signs are absent, neuroimaging is unrevealing, and blood cultures are negative. Rapid CSF molecular testing can provide clinically actionable etiological information at an early stage, while culture and MALDI-TOF MS remain important for microbiological confirmation and susceptibility assessment. The combination of rapid molecular detection with conventional microbiological confirmation may therefore facilitate earlier recognition of *L. monocytogenes* CNS infection and more timely transition from empirical to pathogen-directed antimicrobial therapy.

## 4. Conclusions

Neurolisteriosis should be considered in older adults presenting with fever and neurological symptoms, even in the absence of classical immunosuppression or prominent meningeal signs. Negative blood cultures and normal or nonspecific neuroimaging findings do not exclude *L. monocytogenes* central nervous system infection. CSF culture remains important for microbiological confirmation and antimicrobial susceptibility assessment, while rapid multiplex molecular testing can provide timely etiological information and facilitate early optimization of antimicrobial therapy. In this case, the complementary use of molecular testing, culture, and MALDI-TOF mass spectrometry enabled rapid and reliable diagnosis and supported timely transition to targeted ampicillin-based therapy. Early recognition and appropriate pathogen-directed treatment remain essential for improving outcomes in this potentially life-threatening infection.

## Figures and Tables

**Figure 1:**
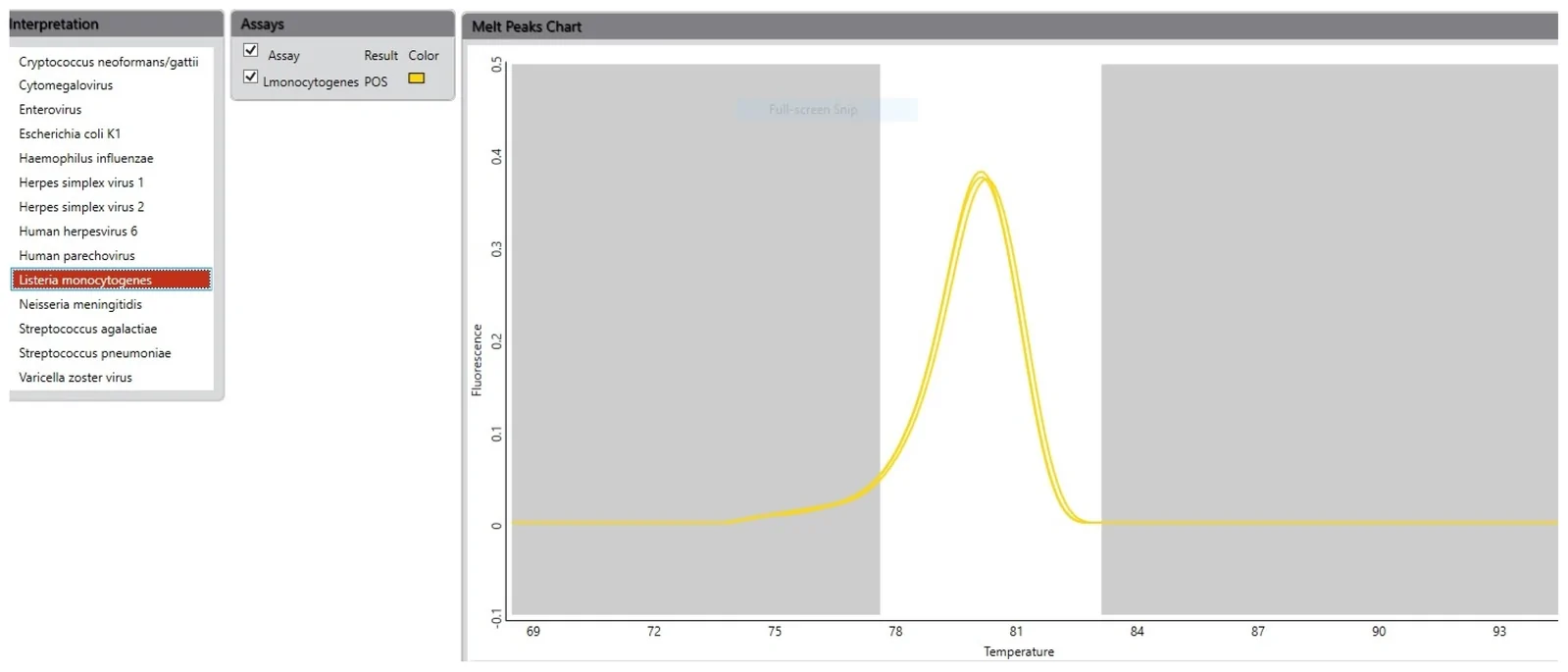
Detection of *Listeria monocytogenes* in cerebrospinal fluid using the BioFire® FilmArray® Meningitis/Encephalitis Panel. The assay demonstrated a positive melt peak consistent with the detection of *L. monocytogenes*.

**Figure 2:**
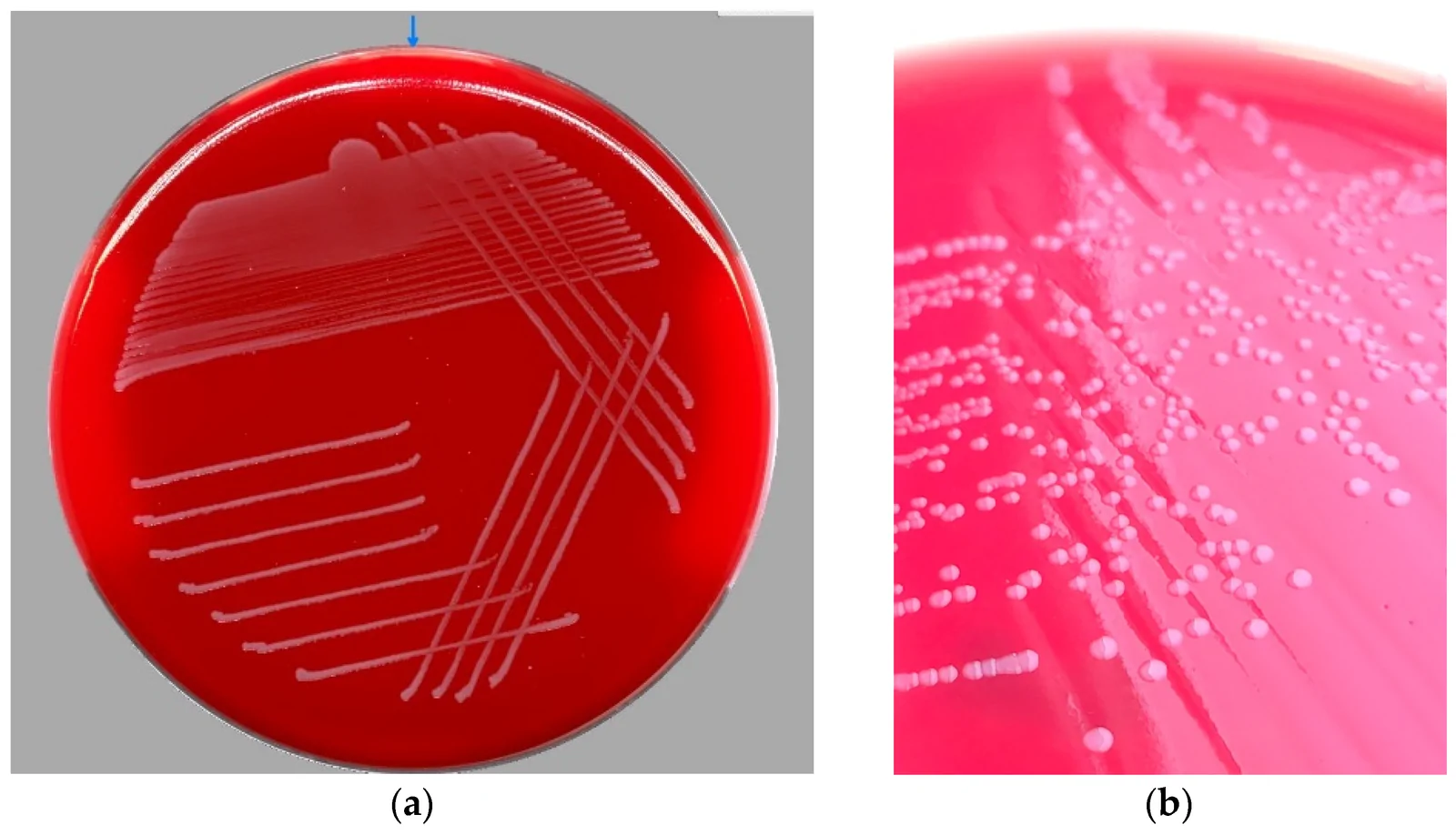
Growth of *Listeria monocytogenes* on Columbia agar. The cerebrospinal fluid sample was inoculated onto Columbia agar, Chocolate agar, and Sabouraud Dextrose Agar and incubated at 35 °C for 18–24 h; Chocolate agar was incubated in 5% CO_2_. After 18 h of incubation, small, smooth, round, translucent colonies measuring approximately 1–2 mm in diameter and surrounded by a narrow zone of β-hemolysis were observed on Columbia agar. (**a**) Colony morphology after incubation; (**b**) isolated colonies showing a narrow zone of β-haemolysis.

**Figure 3:**
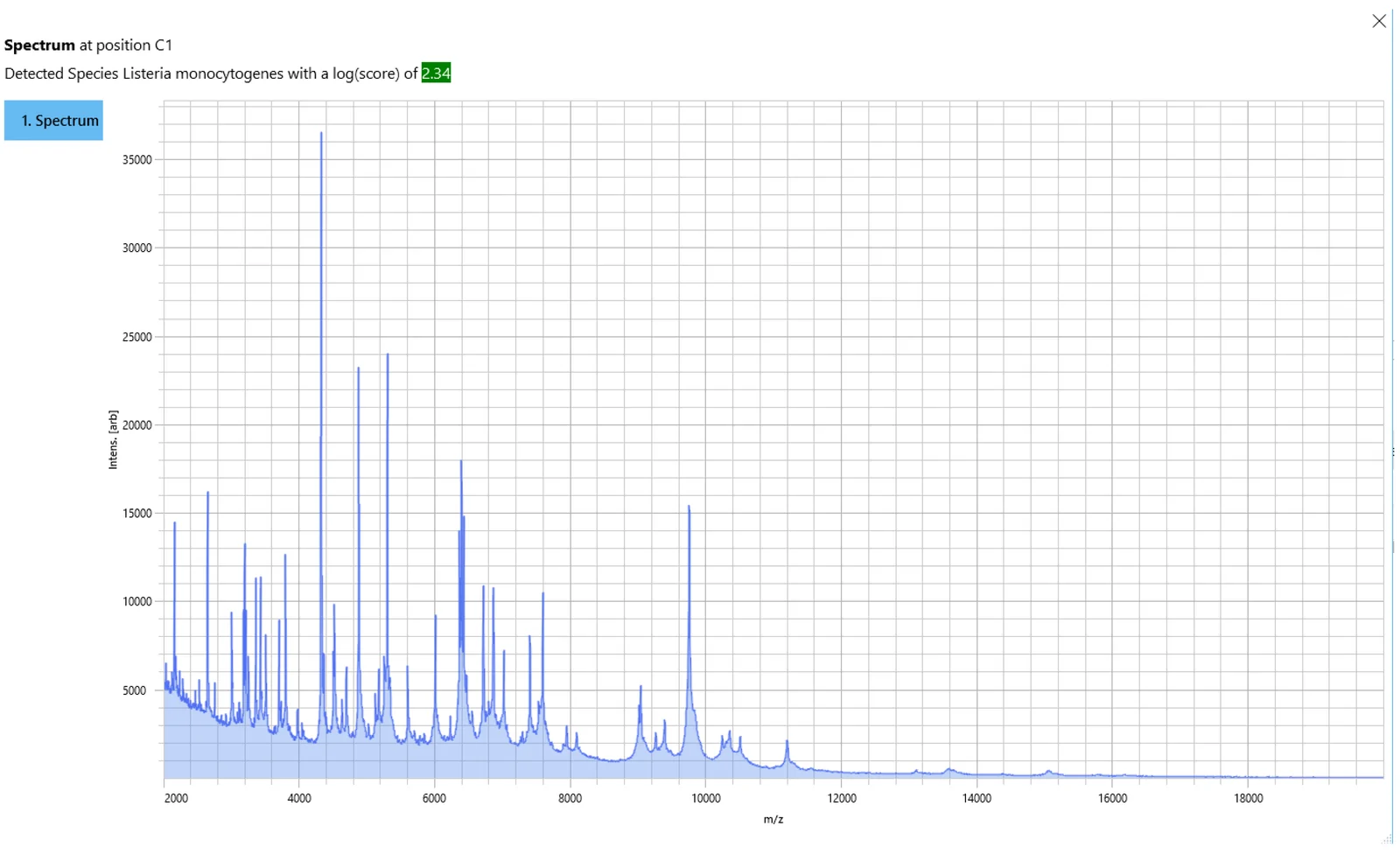
MALDI-TOF mass spectrum of *Listeria monocytogenes*. Colonies grown on Columbia agar were identified using the MALDI Biotyper Sirius system (Bruker). A Bacterial Test Standard was used for run validation, and an identification score of 2.34 was obtained.

**Figure 4:**
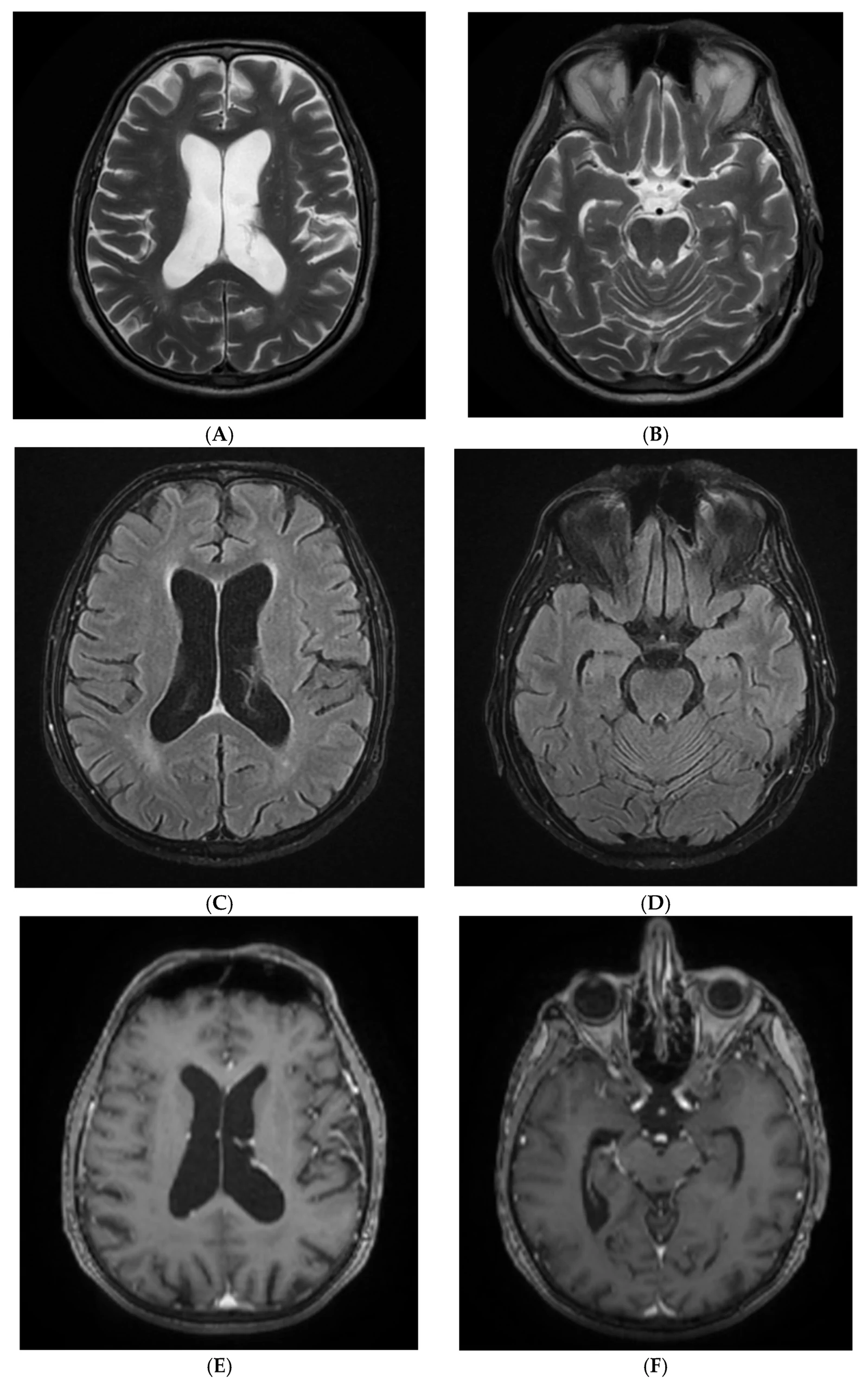
Brain MRI findings. Representative axial T2-weighted PROPELLER images (**A**,**B**), T2-FLAIR images (**C**,**D**), and T1-weighted images (**E**,**F**). The images show several small periventricular hyperintense white-matter lesions and periventricular leukoaraiosis, consistent with chronic microvascular changes. No ventricular enlargement suggestive of hydrocephalus, brainstem or cerebellar abnormalities, or other acute intracranial pathology is identified.

**Table 1:** Dynamics of systemic inflammatory and laboratory parameters during hospitalization, with corresponding reference ranges.

Parameter	Day 1	Day 2	Day 5	Day 10	Day 18	Reference Range
WBC, ×10^3^/µL	**10.76**	9.08	7.99	—	**11.31**	4–10
Neutrophils, ×10^3^/µL	**10.01**	**8.04**	5.66	—	**9.71**	1.5–7
Neutrophils, %	**93.0**	**88.6**	**70.9**	—	**85.9**	50–70
Lymphocytes, ×10^3^/µL	**0.32**	**0.52**	1.76	—	1.26	0.8–4
Lymphocytes, %	**3.0**	**5.7**	22.0	—	**11.1**	20–40
CRP, mg/L	**165**	**219.7**	—	**22.4**	1.1	0–5
ESR, mm/h	—	**80**	**20**	—	—	3–10
Fibrinogen, mg/dL	—	**490**	327	—	—	200–400
Creatinine, mg/dL	0.72	0.74	**0.56**	**0.61**	0.73	0.7–1.3

Values outside the reference range are shown in bold. Abbreviations: CRP, C-reactive protein; ESR, erythrocyte sedimentation rate; WBC, white blood cell count; —, not measured or unavailable.

**Table 2:** Cerebrospinal fluid findings at diagnosis and during follow-up, with corresponding reference values.

Parameter	Day 2	Day 16	Reference Values
Appearance	Clear	Clear, transparent	Clear, transparent
Total cell count, cells/µL	**320**	**68**	0–5
Neutrophils, cells/µL	**176 (55%)**	**52 (76.5%)**	—
Lymphocytes, cells/µL	**144 (45%)**	0 (0%)	—
Red blood cells/µL	0	16	0–5
CSF glucose, mg/dL	**28**	63	40–70
Concomitant serum glucose, mg/dL	105	—	82–115
CSF protein, mg/dL	**158.6**	**53.5**	15–45
CSF chloride, mmol/L	**113**	**117**	118–132

Values outside the reference range are shown in bold. Abbreviations: CSF, cerebrospinal fluid. —, not available.

**Table 3:** Timeline of the patient’s clinical course, diagnostic investigations, treatment, and clinical evolution.

Day	Clinical Condition	Diagnostic Investigations	Treatment/Results
Day 1	Clinical presentation at admission: fever, bradylalia, neck stiffness, mildly positive Kernig I and II signs, positive Romberg test, conscious and fully oriented	Initial clinical assessment	Empirical antimicrobial therapy initiated
Day 2	Persistent neurological symptoms	CSF collected	Vancomycin initiated
Day 3	Afebrile, persistent neurological symptoms	FilmArray® performed; *L. monocytogenes* detected	Ampicillin initiated
Day 5	Persistent neurological symptoms, hiccups, walking difficulty	CSF culture finalized, *L. monocytogenes* identified by MALDI-TOF MS	*L. monocytogenes* confirmed
Day 8	Persistent neurological symptoms, hiccups, walking difficulty	GeneXpert MTB/RIF performed on CSF	*Mycobacterium tuberculosis* DNA not detected
Day 11	Persistent neurological symptoms, brady psychic persistent hiccups and increasing difficulty walking	Brain MRI performed	Periventricular microvascular white-matter lesions and leukoaraiosis, without pathological enhancement, diffusion restriction, or other acute intracranial abnormalities
Day 16	Clinical improvement	Repeat CSF evaluation	Clear CSF, with marked improvement in inflammatory parameters and no microbial growth on repeat culture

Abbreviations: CSF, cerebrospinal fluid; GeneXpert MTB/RIF, GeneXpert *Mycobacterium tuberculosis*/rifampicin; MRI, magnetic resonance imaging; DNA, deoxyribonucleic acid; MALDI-TOF MS, matrix-assisted laser desorption/ionization time-of-flight mass spectrometry.

## Data Availability

The data supporting the findings of this case study are available from the corresponding author upon reasonable request. The data are not publicly available due to privacy and ethical restrictions regarding patient medical records.

## Abbreviations

The following abbreviations are used in this manuscript:

*L. monocytogenes*	*Listeria monocytogenes*
CSF	Cerebrospinal fluid
HIV	Human immunodeficiency virus
ECDC	European Centre for Disease Prevention and Control
EU/EEA	European Union/European Economic Area
CNS	Central Nervous System
NYHA	New York Heart Association
CT	Cranial Computed Tomography
MALDI-TOF MS	Matrix-assisted Laser Desorption/Ionization Time-of-flight Mass Spectrometry
EUCAST	European Committee on Antimicrobial Susceptibility Testing
MRI	Magnetic Resonance Imaging
CRP	C-reactive protein
ESR	Erythrocyte Sedimentation Rate
WBC	White blood cell count
